# Editorial for the Special Issue Titled “Design of Polymeric Hydrogels Biomaterials”

**DOI:** 10.3390/gels10050344

**Published:** 2024-05-18

**Authors:** Ana Paula Serro, Maria Vivero-Lopez, Diana C. Silva

**Affiliations:** 1 Centro de Química Estrutural, Institute of Molecular Sciences, Instituto Superior Técnico, Universidade de Lisboa, Av. Rovisco Pais, 1049-001 Lisboa, Portugal; anapaula.serro@tecnico.ulisboa.pt; 2 School of Pharmacy, University of Nottingham, University Park, Nottingham NG7 2RD, UK

Hydrogels have attracted great interest in the biomedical applications field in recent years. Their biocompatibility, similarity with biological tissues, and ability to be tailored with specific properties make them one of the most promising groups of biomaterials [1]. They can be manipulated to obtain controlled structures and dynamic functionalities or mimic the biological complexity of live tissues. They can also be produced as films, 3D printable structures, fibers, and nanoparticles, making them quite versatile. Hydrogels can also load different compounds within their polymeric network to be used as delivery platforms capable of providing sustained drug release [2]. Some hydrogels behave as smart stimuli-responsive materials since their network can suffer modifications in response to external triggers (e.g., pH, temperature, electrical and magnetic fields, light, or the presence of different biomolecules) and change their hydrophilicity, swelling capacity, physical properties, or molecule permeability [3]. They also present self-healing or shape memory properties. Hydrogels currently have a large number of applications that range from the ophthalmic area (contact lenses, intraocular lenses, and ocular implants) to the cardiovascular (catheter coatings and valves) or skin healing/substitution area (suture threads, wound dressings, and skin grafts). Their role has also become increasingly important in areas such as tissue engineering and regenerative medicine (where they can be used as cell scaffolds) and biosensing [4].

Although we have made several advances in the design of hydrogels for biomedical applications in recent years, many challenges remain in obtaining new materials that, in addition to being safe, ensure an efficient performance. This Special Issue brings together six original research works and five reviews focusing on the most diverse topics related to this theme. Examples of new materials intended for soft tissue augmentation, scaffolds, or drug delivery can be found. Reviews addressing specific hydrogel applications, such as the treatment of bladder cancer or the production of microneedles, and current issues of great interest, like the use of artificial intelligence (AI) for hydrogel development, are presented. Some of the most recent achievements in the area illustrating different aspects of the synthesis, characterization, and application of this type of materials that will certainly continue at the forefront of biomaterial applications are also shown.

Della Salla et al. developed a composite hydrogel based on hyaluronic acid (HA)/carboxymethyl cellulose (CMC), crosslinked with 1,4-butanediol diglycidyl ether (BDDE), intending it to be used in soft tissue augmentation. To obtain a good-performing HA-based hydrogel filler, they prepared materials with different HA/CMC ratios and reaction conditions (different polymerization temperatures during different times) and evaluated their viscoelastic properties, thermal stability upon sterilization in an autoclave, and swelling capacity. The hydrogel containing HA/CMC at a ratio of 1/1, which was prepared at room temperature for 24 h, presented the highest viscoelastic moduli before and after thermal treatment. It showed a dense crosslinking network that explained its rheological properties and thermal resistance. In tests carried out with fibroblasts, the hydrogels led to a cell viability of 90%, and there were no significant changes in cell morphology.

In another study, Lim et al. developed a new strategy to design multi-layered hydrogels for soft hydrogel actuators. They studied the effect of using diffusion to produce an interfacial layer between each layered hydrogel on the enhancement of the design and fabrication precision. The presence of this interfacial layer reduced the degree of mismatch in the self-folding process. The results show a direct relation between the interfacial layer’s thickness and its curvature radius during the self-folding process of the multi-layered hydrogel. Such a layer ensures the integrity of the system in operation as it prevents the separation of layers in the multi-layered hydrogel during actuation.

Gialouri et al. grafted a sodium alginate-based copolymer using thermoresponsive poly(*N*-isopropylacrylamide) (PNIPAM) chains and combined it with methylcellulose (MC) to be used in scaffolds. The material was achieved via a dual crosslinking mechanism including ionic interactions among Ca^2+^ and carboxyl groups and secondary hydrophobic associations of PNIPAM. The results demonstrate that MC significantly enhanced the mechanical properties. The dynamic moduli of the resulting gels make them suitable for the 3D printing of scaffolds. Adhered pre-osteoblastic cells showed a high viability promoting osteogenic potential, as evidenced by the increased alkaline phosphatase activity, calcium, and collagen production.

Carboxymethyl chitosan (CMCh) microgels were synthesized by Sahiner and coworkers with a tailored size and zeta potential for drug delivery purposes by using a microemulsion environment and divinyl sulfone (DVS) as a crosslinker. The microgels presented a spherical structure and a size in the range of 1–10 µm. The materials showed high biocompatibility in cytotoxicity tests with L929 fibroblasts. The antibiotic drug Vancomycin (Van) was used as the model drug to verify its drug-carrying abilities. The MIC values of the drug released from the Van@CMCh microgels after 24 h were 68.6 and 7.95 µg/mL for *E. coli* and *S. aureus*, respectively. The results demonstrate that Van@CMCh microgels have an effective antibiotic effect against *S. aureus* up to 72 h.

In turn, a lignocellulose sponge containing pentoxifylline (PTX)-loaded lecithin/chitosan nanoparticles (LCNs) was developed by Dehghani et al. to be used in wound dressings. They functionalized lignocellulose hydrogels by oxidation/amination, freeze-dried them, and loaded them with nanoparticles. Drug release assays showed that PTX was released in a sustained way. In vivo wound healing studies were performed in rats to which full-thickness excisional wound models were induced. Histological examination confirmed that the PTX-loaded hydrogels performed better and were more suitable for treating chronic wounds compared to the unloaded hydrogels and those that underwent normal treatment with saline solution.

Polyglycerol dendrimers (PGDs) have demonstrated remarkable properties for drug delivery and solubilization, bioimaging, and diagnostics. Ooya and Lee produced PGD hydrogels crosslinked with ethylene glycol diglycidylether (EGDGE) at various concentrations and evaluated their potential for controlling the release of poorly soluble drugs. Paclitaxel (PTX), an anti-cancer drug, was loaded by soaking hydrogels in the drug solution. The increase in the swelling capacity enhanced PTX loading. No evidence of PTX crystallization was observed as the hydrogels remained transparent, and an FTIR analysis revealed a good dispersion of the drug. About 60% of the loaded PTX was released in sink conditions within 90 min. The results show the potential of these hydrogels for the fast release of hydrophobic drugs, e.g., for oral administration.

In their review, Sghier et al. gathered information on the latest advances in the development of nanoemulgels for wound healing, skin appendage infections, inflammatory skin diseases, skin cancer, neuropathy, or anti-ageing purposes, encapsulating a wide range of molecules, including commercial drugs, repurposed drugs, and other natural and synthetic compounds. All developed formulations showed more advantageous characteristics than those that are currently marketed, with adequate droplet size, PDI, pH, stability, viscosity, spreadability, drug release, and drug permeation and/or retention capacity. Their safety and efficacy were confirmed in vitro and/or in vivo, demonstrating their potential to be used as platforms to replace current therapies or as potential adjuvant treatments, which can one day effectively reach the market to help combat high-incidence skin or systemic diseases and conditions.

Cutting-edge topics, including artificial intelligence (AI) and machine learning (ML), were also addressed. Negut et al. explored their integration in hydrogel development, highlighting their importance in improving the design, characterization, and optimization of hydrogels for various applications. The concept of using AI for train hydrogel design is introduced, underlining its potential to decipher intricate relationships between hydrogel compositions, structures, and properties from complex datasets. Classic physical and chemical techniques in hydrogel design are described to lay the foundation for advances in AI/ML along with AI/ML-enhanced numerical and analytical methods. ML techniques, such as neural networks and support vector machines, which accelerate pattern recognition and predictive modeling using large datasets and advance the discovery of hydrogel formulations, are also presented. In sum, this review shows how AI and ML have transformed hydrogel design by accelerating material discovery, optimizing properties, reducing costs, enabling precise customization, and offering innovative solutions for drug delivery, tissue engineering, wound healing, and more.

Hydrogels have also gained attention in the field of transdermal microneedles thanks to their tunable properties, which allow them to be exploited as delivery systems and extraction tools. However, since hydrogel microneedles are a new emerging technology, their manufacture faces several challenges that need to be addressed for them to be redeemed as a viable pharmaceutical option. Shriky et al. reviewed hydrogel microneedles from a material standpoint, independent of their mechanism of action, citing advances in their formulation, presenting relevant manufacturing and characterization methods, and discussing the regulatory and manufacturing challenges faced by these emerging technologies before their approval.

In their review, Omidian et al. emphasize the adaptability and promise of injectable hydrogel nanocomposites in biomedical research. Injectable hydrogels have become popular due to their ability for controlled release, targeted delivery, and improved mechanical properties. These materials exhibit potential in many areas, including joint ailments, cardiac regeneration, eye disease treatment, and post-operative analgesia. They are also useful in tissue regeneration, cardiovascular issues, ischemic brain injury, and personalized cancer immunotherapy. Moreover, nano-hydroxyapatite-enriched hydrogels offer promise in bone regeneration, tackling bone defects, osteoporosis, and tumor-associated recovery challenges. In wound care and cancer treatment, they facilitate controlled release, expedite wound healing, and target drug release. Their review also includes a perspective section that delves into future possibilities, underscores interdisciplinary collaboration, and emphasizes the bright prospects of injectable hydrogel nanocomposites in biomedical research and applications.

The last review, prepared by Lima et al., focuses on describing the current situation of bladder cancer, the tenth most common type of cancer worldwide. After describing the disease and available treatments, they present a report on the main mucoadhesive polymer-based drug delivery systems (DDSs) that were developed in recent years. These DDSs have an increased ability to improve the drug residence time, permeation capacity, and target release, which may prevent the need for frequent catheter insertions with reduced intervals between doses that are followed by current intravesical therapies and which are highly demotivating for patients. A brief review of the methods used for assessing mucoadhesion properties is also shown, along with a discussion of the different polymers suitable for this application.
